# The Effects of Seven-Day Exposure to Silver Nanoparticles on Fertility and Homeostasis of Zebrafish (*Danio rerio*)

**DOI:** 10.3390/ijms231911239

**Published:** 2022-09-24

**Authors:** Hubert Szudrowicz, Maciej Kamaszewski, Antoni Adamski, Marek Skrobisz, Justyna Frankowska-Łukawska, Maciej Wójcik, Joanna Bochenek, Kacper Kawalski, Jakub Martynow, Patryk Bujarski, Pola Pruchniak, Ewelina Latoszek, Paweł Bury-Burzymski, Adrian Szczepański, Sławomir Jaworski, Arkadiusz Matuszewski, Andrzej Przemysław Herman

**Affiliations:** 1 Institute of Animal Sciences, Warsaw University of Life Sciences, Warsaw, Ciszewskiego 8, 02-786 Warsaw, Poland; 2 Institute of Biology, Warsaw University of Life Sciences, Warsaw, Nowoursynowska 159, 02-776 Warsaw, Poland; 3 The Kielanowski Institute of Animal Physiology and Nutrition, Polish Academy of Sciences, Instytucka 3, 05-110 Jabłonna, Poland; 4 International Institute of Molecular and Cell Biology, Księcia Trojdena 4, 02-109 Warsaw, Poland

**Keywords:** genotoxicity, gonads, histology, liver, silver nanoparticles, zebrafish

## Abstract

Silver nanoparticles (AgNPs) are found in open waters, but the effect of their low concentrations on an organism’s homeostasis is not fully understood. The aim of the study was to determine the short-term exposure effects of AgNPs coated by PvP (polyvinylpyrrolidone) on the homeostasis of livers and gonads in zebrafish. Sexually mature zebrafish were exposed for seven days to silver ions (0.01 mg/dm^3^) or AgNPs (0.01; 0.05; 0.1; 0.5; 1.0 mg/dm^3^). On the last day, the liver, testes, and ovaries were subjected to a histology analysis. In the liver, we analyzed the expression of the *cat*, *gpx1a*, *gsr*, *sod1*, and *cyp1a* genes. On the last day of the experiment, the lowest survival rate was found in the AgNPs 0.05 mg/dm^3^ group. The histological analysis showed that AgNPs and silver ions cause an increase in the area of hepatocytes. The highest proliferation index of hepatocytes was found in the AgNP 0.05 mg/dm^3^ group. Furthermore, AgNPs were found to interfere with spermatogenesis and oogonesis as well as reduce the expression levels of the *cat*, *gpx1a*, and *sod1* genes in the liver compared with the control group. Based on the results, it can be concluded that exposure to AgNPs causes cytotoxic changes in zebrafish, activates the immune system, negatively affects the process of meiosis in the gonads, and generates oxidative stress.

## 1. Introduction

A new branch of science has emerged over the past few decades, which focuses on inorganic nanoparticles whose structures exhibit different physical, chemical, and biological properties than their macro-equivalents. The technical and medical applications of nanoparticles are numerous, and new ideas are being developed almost daily. Silver has been used for years in medicine due to its well-known antimicrobial properties [1]. The miniaturization of silver particles has allowed for the obtaining of new types of materials that can be used in everyday human life. These particles are commonly applied in such products such as electronics, paints, cosmetics, household machines, clothes/fabrics, as well as in food technology and medical purposes [2,3].

The effects of nanotechnology products on organisms inhabiting aquatic ecosystems have been analyzed for many years. Nanotoxicological work focuses on assessing the toxicity of nanoparticles commonly used by humans, such as silver nanoparticles, titanium dioxide nanoparticles, copper nanoparticles, zinc oxide nanoparticles, and graphene nanoparticles, on aquatic invertebrates (e.g., *Daphnia* sp.) [4] or fish such as the zebrafish (*Danio rerio*), medaka (*Oryzias latipes*), Siberian sturgeon (*Acipenser baerii*), and rainbow trout (*Oncorhynchus mykiss*) [5,6,7,8,9]. Most of the studies were conducted under laboratory conditions, where nanoparticles were used at concentrations that induce toxic changes rather than at concentrations that have been observed or predicted (modeled/predicted) in aquatic environments. Often, the nanoparticles analyzed in studies oscillate at concentrations that can cause sublethal or lethal effects. As Dumont et al. [10] and Syafiuddin et al. [11] pointed out, the environmental concentrations of commonly used nanoparticles are far lower than those commonly studied. Therefore, it is necessary to analyze the effects of silver nanoparticles on aquatic organisms in the concentration range of 0.03–0.32 µg/dm^3^ of silver nanoparticles in aquatic ecosystems [12]. The model species zebrafish (*Danio rerio*), which belongs to the family *Danionidae* (formerly *Cyprinidae*), is currently the most widely used species in laboratory studies that involve, among other things, toxicology or biomedical research. With this in mind, zebrafish are often used as a research model in studies analyzing the toxicity of nanoparticles.

Another issue to pay attention to when analyzing the toxicity of nanoparticles is their impact on humans and the environment at various stages, from their synthesis to their use and disposal. As McGillicuddy et al. [13] suggested, the widespread use of products containing silver nanoparticles poses risks to human health and the environment. Many of these contaminants are believed to end up in both soil and aquatic ecosystems, where silver nanoparticles can accumulate in the tissues of terrestrial and aquatic organisms, causing toxic effects. In addition, the effects of various nanoparticles on gonadal development and animal fertility are also poorly understood. Few studies have been primarily conducted on model species. Ong et al. [14] showed that silver nanoparticles disrupt spermatogenesis and reduce fertility in the *Drosophila melanogaster*. Moreover, it is known that silver nanoparticles can also negatively affect the female reproductive system by inducing oxidative stress and stimulating apoptosis of ovarian follicle cells in zebrafish (*Danio rerio*) [15] and by affecting ovarian steroidogenesis in hens (*Gallus gallus domesticus*) [16].

Therefore, it is necessary to conduct studies that show how commonly used nanoparticles, in concentrations comparable to those expected in the environment, can disrupt the homeostasis of aquatic organisms and affect the demographics of their populations. In addition, some of these studies do not address the commonly used nanoparticles that are conjugated (coated) with substances to stabilize their physicochemical properties, such as citrate or polyvinylpyrrolidone (PvP). Therefore, the aim of this study was to determine the effects of silver nanoparticles coated by PvP at concentrations similar to those found in aquatic ecosystems on the liver homeostasis, germinal cell formation in the gonads, and genotoxicity of zebrafish during short-term exposure.

## 2. Results

**Figure 1:**
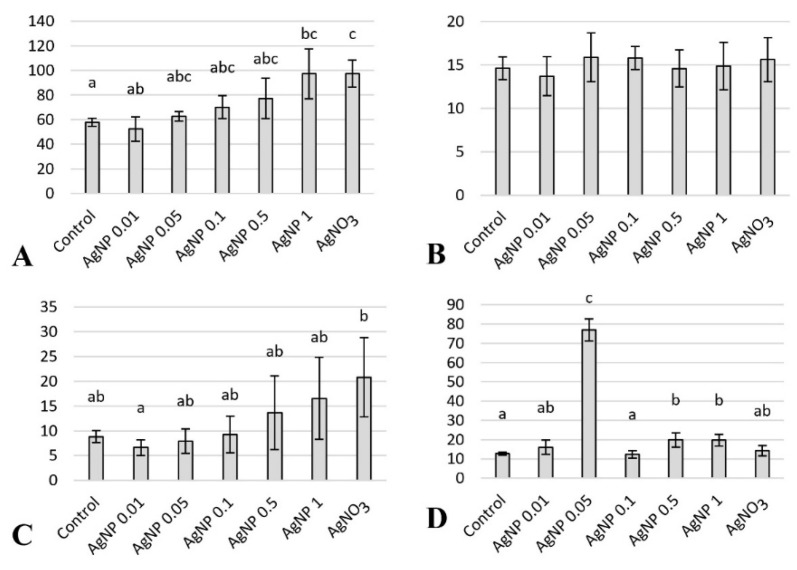
The histomorphometric parameters of fish livers on the last day of the experiment: (**A**) area of hepatocytes (µm^2^); (**B**) area of hepatocytes nuclei (µm^2^); (**C**) area of lipid vacuoles in hepatoctes (µm^2^); and (**D**) Percentage (%) of PCNA+ nuclei of hepatocytes. Mean values ± SD are shown. Different letters indicate the statistical differences between groups.

**Figure 2:**
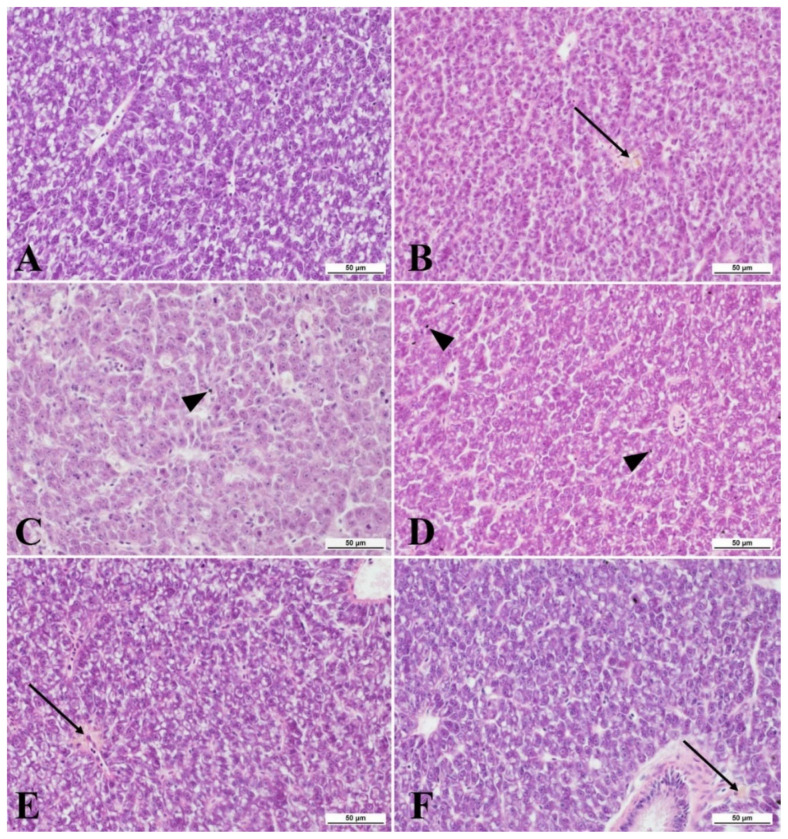
A cross-section of zebrafish livers from the control group (**A**) and when exposed to: (**B**) AgNP solution at 0.01 mg/dm^3^; (**C**) AgNPs at 0.05 mg/dm^3^; (**D**) AgNPs at 0.1 mg/dm^3^; (**E**) AgNP at 1.0 mg/dm^3^; and (**F**) AgNO_3_ at 0.01 mg/dm^3^. Arrow: melanomacrophage centers. Arrowhead: macrophages with nanoparticle deposition. HE staining performed. Scale bars 50 µm.

There was a one-week exposure of zebrafish to nanoparticles at concentrations of 0.01, 0.5, and 1.0 mg/ dm^3^ as well as silver ions. The control group the survival rate was 100%. The lowest survival rate was found in the group of fish exposed to silver nanoparticles at a concentration of 0.05 mg/dm^3^ (88.66 ± 15.27%). The AgNPs 0.1 mg/dm^3^ group also had a lower survival rate (96.66 ± 5.77%) compared with the control group. However, there were no differences in the survival rates of the fish. In the group of fish not exposed to the tested xenobiotics, polygonal hepatocytes with an average area of 57.8 ± 3.23 µm^2^ were found on sections in the liver parenchyma (Figure 1 and Figure 2). Lipid vacuoles with an average area of 8.82 ± 1.22 µm^2^ were found in the cytoplasm of the cells (Figure 1). Hepatocytes in the parenchyma were arranged in a regular pattern along the sinusoids and bile ducts. Single macrophages in the sinusoids were visible, but the formation of melanomacrophage centers were not observed (Figure 2).

### 2.1. Histology of Liver

The hepatocytes with the smallest surface area and the smallest cytoplasmic area occupied by lipid vacuoles were found in the AgNP 0.01 mg/dm^3^ experimental group, and the values of these parameters were statistically significantly different from those found in the fish from the AgNO_3_ 0.01 mg/dm^3^ group, where they were the highest (Figure 1 and Figure 2). Compared with the control group, higher, statistically significant hepatocyte areas were observed in the groups of fish exposed to silver nanoparticles at the concentration of 1.0 mg/dm^3^ and in the AgNO_3_ 0.01 mg/dm^3^ group (Figure 1). The average cross-sectional area of the hepatocyte nuclei was not significantly different between the experimental groups (Figure 1).

The highest proliferation index of hepatocyte nuclei in the liver parenchyma was found in the liver of fish in the AgNP 0.05 mg/dm^3^ group, while the lowest was found in the AgNP 0.1 mg/dm^3^ group; the differences were statistically significant (Figure 1). In the experimental groups where fish were exposed to the highest concentrations of silver nanoparticles (the AgNP 0.5 and 1.0 mg/dm^3^ groups), a statistically significant higher proliferation index of hepatocyte nuclei in the liver was detected compared with the control group (Figure 1).

In the livers of fish exposed to nanoparticle solutions and silver ions, the appearance of sinusoidal macrophages and melanomacrophages was observed (Figure 2). The number of melanomacrophages in the sinusoids increased, causing the formation of melanomacrophage centers; these formations were observed in all experimental groups (Figure 2). In fish exposed to silver nanoparticles at concentrations of 0.05 and 0.1 mg/dm^3^, deposition of silver nanoparticles was observed in the cytoplasm of the macrophages infiltrating the liver parenchyma (Figure 2C,D).

### 2.2. Ultrastructure of Hepatocytes

**Figure 3:**
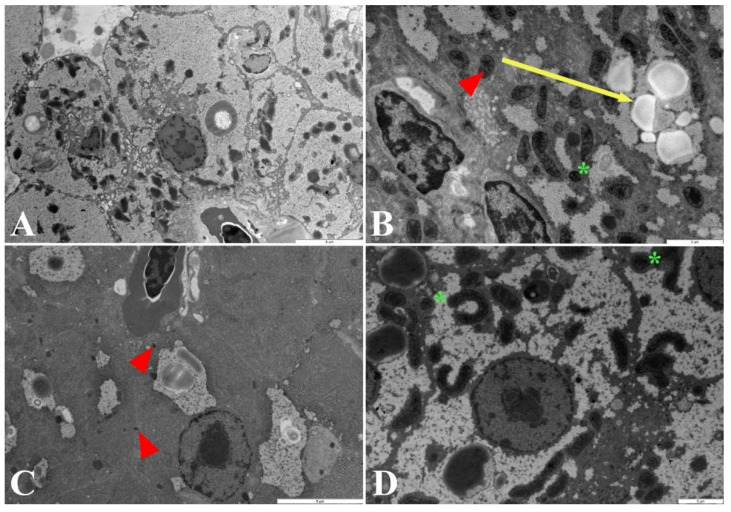
Ultrastructure of zebrafish hepatocytes from the control group (**A**) and when exposed to: (**B**) AgNPs at 0.05 mg/dm^3^, (**C**) AgNPs at 1.0 mg/dm^3^, (**D**) AgNO_3_ at 0.01 mg/dm^3^. Arrow: myelin bodies. Arrowhead: nanoparticle deposition. * Mitochondria with an abnormal shape. Scale bars in photo (**A**,**C**): 5 µm; in photo (**B**,**D**): 2 µm.

The analysis of the liver from the control group showed a normal organelle ultrastructure: a rounded nucleus; numerous oval and elongated mitochondria, extensive endoplasmic reticulum, lipid droplets, and vast fields occupied by glycogen particles (Figure 3). In fish exposed to silver nanoparticles, observations under the electron microscope showed cytopathological changes in the liver tissue compared with the control group. The most visible was the disorganization of the cell structure, changes in the shape of the mitochondria and nucleus, and a significant loss of glycogen. These alterations intensified as the concentration of nanoparticles increased. In the liver parenchyma of fish exposed to nanoparticles at concentrations of 0.05 mg/dm^3^ and above, we observed the formation of myelinous bodies (Figure 3B), which are membranous phagolysosomes; they were particular numerous in the livers of fish exposed to silver nanoparticles at the concentration of 1.0 mg/dm^3^. In addition, the deposition of black electron-dense spots was found, which may indicate deposits of silver nanoparticles in the cell cytoplasm (Figure 3C). In the cytoplasm of fish cells exposed to silver ions (the AgNO_3_ group), we observed the area occupied by glycogen and lipid drops. Regarding pathological changes, we only observed abnormal shapes of mitochondria (Figure 3D).

### 2.3. Histology of the Ovary

**Figure 4:**
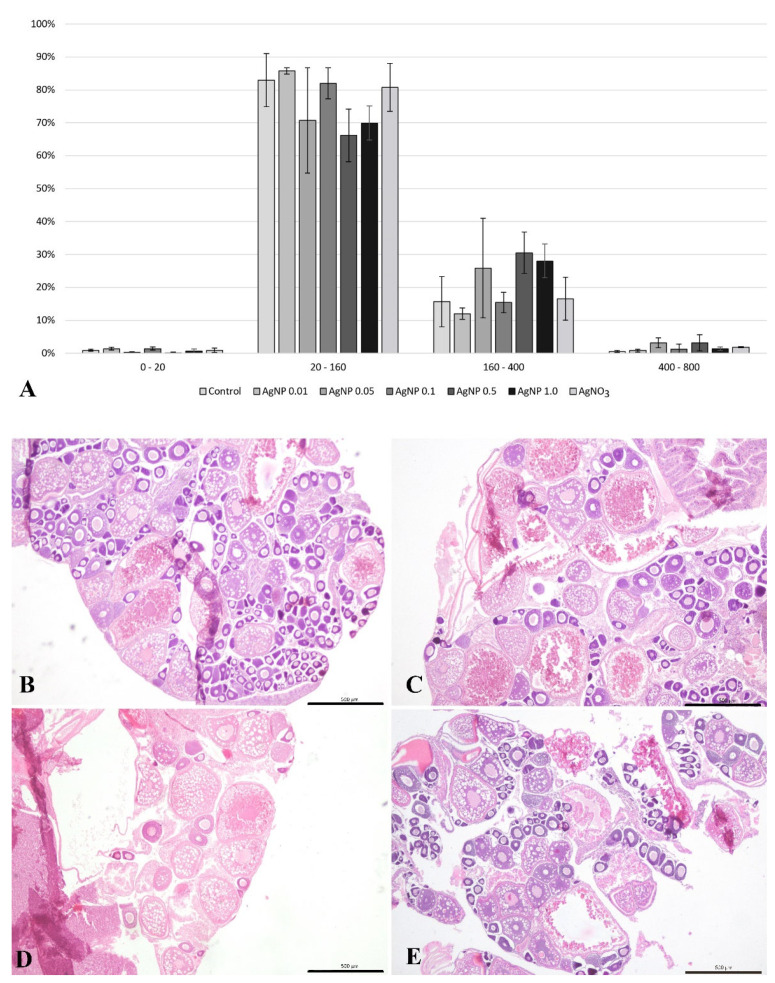
Distribution of oocyte diameter (mean values ± SD) of zebrafish from all experimental groups (**A**). Cross-section through the female gonad of zebrafish from the experimental groups: (**B**) control group, (**C**) AgNPs at 0.01 mg/dm^3^, (**D**) AgNPs at 1.0 mg/dm^3^, and (**E**) AgNO_3_ at 0.01 mg/dm^3^. HE staining performed. Scale bars: 500 µm.

The histological analysis of the ovaries showed no pathological changes in this organ (Figure 4). Sections of the ovaries of fish from all experimental groups showed the presence of oogonia and oocytes at various stages of development. Oocytes of the 20–160 µm diameter class were the most abundantly represented (Figure 4). However, the histomorphometric analysis based on the analysis of oocyte diameter distribution showed that in ovaries of fish exposed to silver nanoparticles at concentrations of 0.5 and 1.0 mg/dm^3^, the proportion of oocytes at the early stages of development was about 70% and was lower compared with the other groups studied (Figure 4).

### 2.4. Histology of the Testis

**Figure 5:**
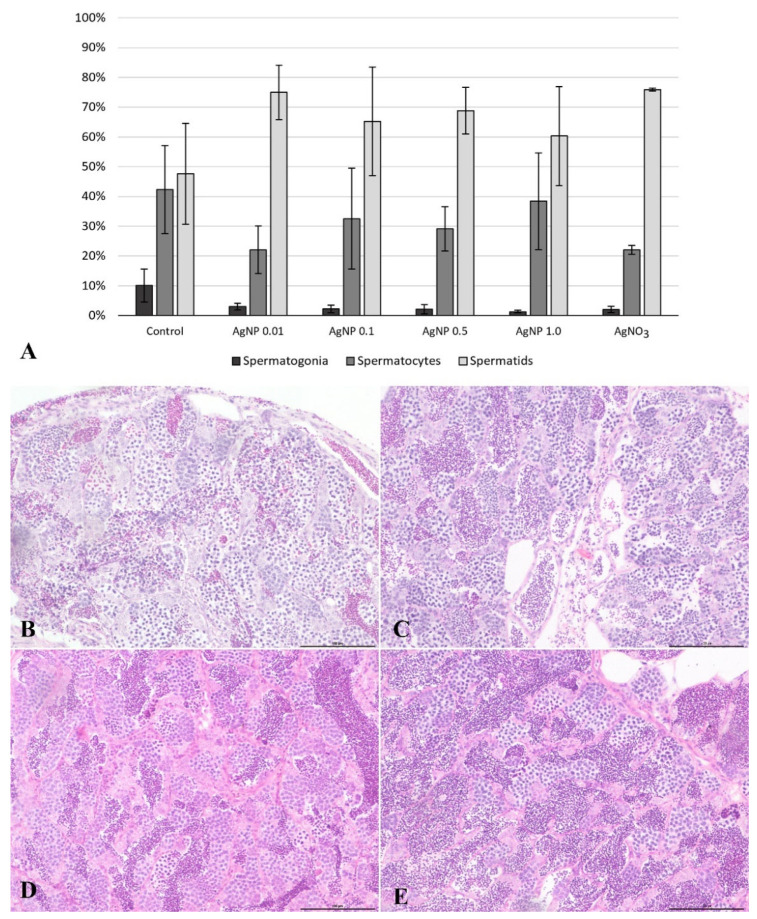
Percentage (mean values ± SD) of spermatogonia, spermatocytes, and spermatids in the testes of zebrafish from the experimental groups (**A**). The cross-section through the male gonad of zebrafish from the experimental groups: control group, (**B**) AgNPs at 0.01 mg/dm^3^, (**C**) AgNPs at 0.1 mg/dm^3^, and (**D**) AgNO_3_ at 0.01 mg/dm^3^ (**E**). HE staining performed. Scale bars: 500 µm.

On cross-sections through the male gonad of fish from all experimental groups, we observed an anastomosis of the seminiferous tubules with normal structures. Spermatogonia, spermatocytes, and spermatids were observed in the epithelium of the tubules, while spermatids were present in the lumen of the tubules (Figure 5). There were no histopathological changes in testicular morphology.

The histomorphometric analysis of the male gonads showed that in all xenobiotic-exposed groups, the proportion of spermatogonia was lower compared with the gonads of fish from the control group. The proportion of spermatids in zebrafish from the experimental groups was higher than in the control group (Figure 5).

### 2.5. Genotoxicity

**Figure 6:**
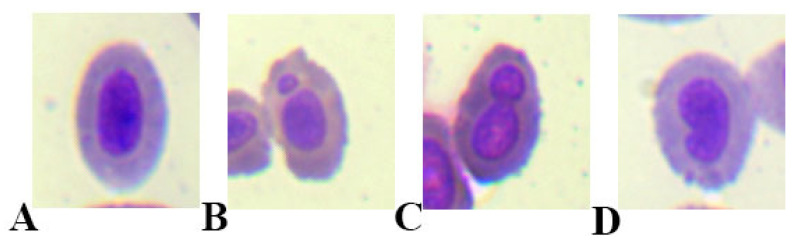
Micronucleus test. Erythrocytes of fish from different groups: (**A**) Control group (normal erythrocyte), (**B**) AgNPs at 1.0 mg/dm^3^ (erythrocyte with micronucleus), (**C**) AgNPs at 0.5 mg/dm^3^ (erythrocyte with segmented nucleus), (**D**) AgNPs at 0.01 mg/dm^3^ (erythrocyte with a kidney shape: reniform). MGG staining performed. Magnification: 1000×.

The analysis of the micronuclei test results showed that there was an increase in the percentage of erythrocytes with micronuclei in the blood smears of fish from the AgNP 1.0 mg/dm^3^ group compared with the other experimental groups (Table 1 and Figure 6). An increase in the percentage of erythrocytes with segmented cell nuclei in the fish blood smears was observed from all the experimental groups except for in the control group and AgNP 0.01 mg/dm^3^ group (Table 1 and Figure 6). The proportion of reniform in erythrocytes was very low and did not differ between the experimental groups (Table 1).

### 2.6. Gene Expression

**Figure 7:**
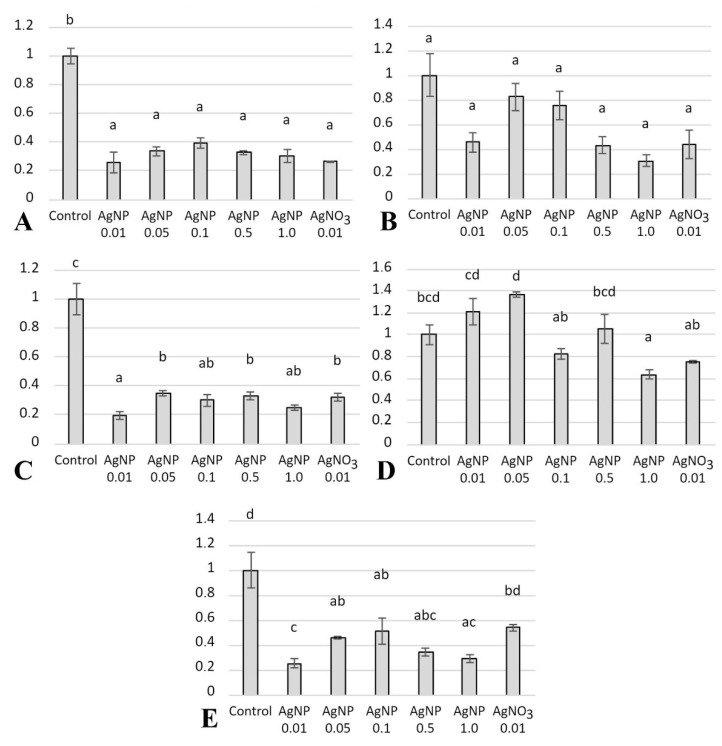
Relative gene expression (mean values ± SEM): (**A**) *cat*; (**B**) *cyp1a*; (**C**) *gpx1a*; (**D**) *gsr*; and (**E**) *sod1* in the livers of the tested zebrafish. Different letters indicate statistical differences between groups.

The gene expression analysis of oxidative stress markers (*cat*, *gpx1a*, *gsr*, and *sod1*) and phase I biotransformation markers (*cyp1a*) in the liver of zebrafish showed changes in the amount of tested transcripts, depending on the concentration of the tested xenobiotic. Exposure of fish to the tested xenobiotics resulted in statistically significant lower expression levels of *cat*, *gpx1a*, and *sod1* in the livers of fish from the tested groups compared with the control group (Figure 7). There were no statistically significant differences in the expression of *cyp1a* in the livers of the tested fish (Figure 7). In contrast, it was found that fish exposed to 0.05 mg/dm^3^ had higher level of *gsr* gene expression compared with the AgNP 1.0 mg/dm^3^ group, and the difference was found to be statistically significant (Figure 7). In the AgNP 1.0 mg/dm^3^ experimental group, *gsr* expression levels were lower than in the control group. The highest level of *gsr* expression was found in the livers of fish from the AgNP 0.05 mg/dm^3^ group, while the lowest level was found in the AgNP 1.0 mg/dm^3^ group, and the differences were statistically significant (Figure 7).

## 3. Discussion

Research assessing the toxicity of silver nanoparticles on fish has been conducted for many years. These studies have included the analyses of the effects of a wide range silver nanoparticle concentrations on survival, bioaccumulation, or toxicity markers. It has been shown that one of the important factors influencing the toxicity of silver nanoparticles is their ability to release ions (AgNO_3_) [17]. It is a complex two-stage process, the intensity of which increases as the surface area of the chemical reaction increases [18]. The surface area of AgNPs is variable depending on the size of nanoparticles and the level of their aggregation. The aggregation area is smaller than the total chemical reaction area of the individual nanoparticles, resulting in reduced toxicity [19]. In the current study, it was observed that despite the use of PvP-coated silver nanoparticles at low concentrations, as similar to those found in the environment, an aggregation of nanoxenobiotics occurs. This phenomenon may explain the non-linear distribution of survival rates in the experiment. In the experiment, the lowest survival rate was found in the group of fish exposed to AgNP at a concentration of 0.05 mg/dm^3^. On the one hand, this may have been due to the fact that silver toxicity may be amplified when PvP-coated AgNPs are used [20]. However, as suggested by Glover et al. [21]**,** it is possible to re-create nanoparticles from silver ions in the environment with a size and coating completely different from the original. In addition, it has been shown that silver nanoparticles to an extent can spontaneously form from dissolved silver ions [22]. The rate of the mentioned phenomena of agglomeration, transition of silver nanoparticles to the ionic state, and their re-formation are usually determined by the type of substance stabilizing the nanoparticles, amount of organic ions and substances, pH, nanoparticle size, salinity, and oxygen concentration in water [23,24]. Polymers such as PvP are also characterized by a lower (than citrate, by an order of magnitude) affinity for binding to proteins. This is an important property, as protein attachment to nanoparticles under physiological conditions greatly limits the bioavailability of AgNPs [25]. Particular attention should also be paid to the mentioned relationship between the size of the nanoparticles themselves and the reaction that they cause; the smaller the silver nanoparticles, the greater the toxicity. This relationship has been observed in studies both on in vitro cultures, in ovo during toxicological tests, in the development of anticancer therapies, and in studies on fungi and bacteria [19,26,27]. Thus, newly formed nanoparticles in experimental or natural environments will not show an identical toxic potential on living organisms.

The most commonly used parameter for assessing the toxicity of nanoparticles is to find the lethal concentration (LC) value. However, the concept of using biomarkers in toxicity assessment is a promising alternative for obtaining early signs of toxicity to avoid further threats to organisms or the environment. Biomarkers are sensitive and specific to the classes of xenobiotics tested. The results obtained during their analysis may be correlated with other toxic effects that are likely to occur at high concentrations [28]. After exposure to nanoparticles in model organisms, they accumulate and decompose in the body. Exposure to sub-lethal doses of nanoparticles changes the biochemical profile before any observed physiological side effects, such as mortality [29,30]. On the other hand, exposure to nanoparticles for several days can also negatively affect the histological structure of organs, including: liver, gills, gonads, disrupting the cell division process [8], and causing genotoxicity.

The observed increased area of hepatocytes, mainly due to the increased area of lipid vacuoles, is a well-known and described marker of the response to stress that is caused by the diet of fish or the presence of heavy and precious metals in the environment [31]. These changes usually indicate a disturbance in the body’s energy and metabolism [32].

In the experiment, an increase in the area of hepatocytes and lipid vacuoles was observed in fish that were exposed to solutions of silver nanoparticles at increasing concentrations, as well as those that were exposed to silver ions. A similar relationship was observed in the liver of rainbow trout exposed to CuNPs [33] and in Siberian sturgeon with lower tested concentrations of AgNPs [7]. The analysis of the proliferation of hepatocyte nuclei showed a non-linear relationship of this biomarker to AgNP concentration, with the highest proliferation index found in fish from the group where there was the lowest survival rate (AgNP 0.05 mg/dm^3^). This may suggest that the repair mechanisms of the liver, the organ responsible for the detoxification of the body, were intensively activated in fish from this particular experimental group. The PCNA protein is known to be involved in DNA replication and repair, which may support the thesis of this polymerase cofactor playing an important role in modulating the response of hepatocytes during an exposure of the organism to xenobiotics. A similar distribution of the proliferative index (with the concentration of silver nanoparticles)—an initial increase in the value of this parameter, and then at the highest concentrations of the tested xenobiotic was observed by [7] in Siberian sturgeon. As the authors suggested, the low level of proliferation in the livers of fish exposed to the highest concentrations of AgNPs may be associated with a decrease in the proliferative potential. Brohi et al. [34] noted that silver nanoparticles, by inducing oxidative stress and mitochondrial dysfunction, lead to the inhibition of cell proliferation. In addition, a number of histopathological changes and ultrastructural abnormalities in the liver indicate that even a seven-day exposure to silver nanoparticle solutions can negatively affect fish homeostasis. The formation of melanomacrophage centers in the liver parenchyma of fish exposed to AgNPs and the accumulation of silver nanoparticles in the cytoplasm of macrophages (especially in the groups with lower survival rates, such as the AgNPs at 0.05 and 0.1 mg/dm^3^ groups) indicate the important role of Browicz–Kupffer cells in the detoxification of the organs from nanoxenobiotic stimuli and the protection of cells from oxidative stress [7,8].

Castellini et al. [35] indicate that nanoparticles can also negatively affect reproductive activity, probably due to their penetration through the blood–testes barrier; they can penetrate germinal tissue. Therefore, the effects of silver nanoparticles on the reproductive system and fertility of model animals are being analyzed. However, to date, there are few studies that have analyzed the in vivo effects of silver nanoparticles on ovaries and oogenesis in fish. In the zebrafish studied, we found that in the ovaries of fish from the AgNP 0.5 and 1.0 mg/dm^3^ experimental groups, an inhibition of meiosis was observed, expressed by a reduced proportion of previtellogenic oocytes and an increased proportion of vitellogenic oocytes. Thanks to the spawning procedure on the last day of the experiment, it is known that the oocytes observed in the gonad were formed during the seven-day exposure to the tested nanoxenobiotics (zebrafish is a model species with continuous and aseasonal reproduction under laboratory conditions). Dayal et al. [36] observed in histopathological changes in the oocytes of ovaries from zebrafish exposed to a chronic 28-day exposure of gold nanoparticles (AuNPs), such as the detachment of zona radiata from the oocyte, appearance of atretic oocytes, and DNA damage in the ovarian cells. The results of this study showed that nanoxenobiotics also induce cytotoxicity and genotoxicity in the female gonad and cause endocrine disruption. Moreover, silver nanoparticles affect the regulation of steroidogenesis-related genes in the ovaries, as shown in studies conducted on fish [20] and chickens [16]. As suggested by Katarzyńska-Banasik et al. [16], the strength of the effect of AgNPs is dependent on the exposure time, which may translate into animal fertility. The results indicate that the long-term exposure of female fish to silver nanoparticles may disrupt the secretion of hormones that regulate oogenesis, which may consequently lead to fertility disorders in the fish population. However, this phenomenon is still poorly understood and requires further experiments to confirm the toxicity of metal nanoparticles on processes within the germinal tissue of the ovary.

The effects of nanoproducts, including silver nanoparticles, on spermatogenesis, sperm motility, and the process of oocyte fertilization are now far better understood. Analyses in various model animal species have shown that silver nanoparticles disrupt the hormones involved in spermatogenesis, induce oxidative stress in the gonad, and induce pathological changes, including AgNP accumulations in the germinal tissue, or decrease sperm motility and viability [12,35,37,38,39]. In addition, Thakur et al. [37] indicated that during a chronic, 90-day exposure of rats to silver nanoparticles, an apoptosis of all germinal cell types in the nucleus occurred, resulting in the depletion of germ cells. Similarly, impaired spermatogenesis and a reduction in the proportion of spermatogonia and spermatocytes in the germinal cells of the testis were observed in the zebrafish tested during a one-week exposure to AgNPs compared with the control group. However, there was no negative effect of silver nanoparticles on the proportion of spermatids, which may be due to the initiation of meiotic divisions which led to the formation of these cells before the experiment. Moreover, Fathi et al. [38] found that silver nanoparticles have an effect on reducing the number of spermatogonia in the male gonad, which was also confirmed by our study. The mechanism of the induced changes in the male gonad during exposure of organisms to silver nanoparticles is not fully known, but as [40] suggested, it may be related to changes in the levels of male sex hormones and the expression of genes responsible for testicular function. In addition, the induction of oxidative stress, cell necrosis and apoptosis, and the crossing of the blood–testes barrier [35], among other processes, by AgNPs also have important effects on the homeostasis of this organ.

The genotoxic effects of nanoproducts have long been analyzed. Silver nanoparticles, which lead to an increase in reactive oxygen species (ROS), have been found to cause oxidative stress, which can consequently lead to DNA damage [41]. During a week-long exposure of zebrafish to silver nanoparticles, no strong genotoxic effects were observed with respect to the tested toxicant. Using the micronucleus test, micronuclei were found to appear only in the blood of fish exposed to the highest concentration of the tested xenobiotic (AgNPs at 1.0 mg/dm^3^). Similar observations were found in the erythrocytes of pacu (*Piaractus mesopotamicus*) by Bacchetta et al. [42]. In contrast, erythrocyte nuclear abnormalities (ENA) were observed in the AgNP 0.5, 1.0, and AgNO3 0.01 mg/dm^3^ groups. As Asare [43] suggested, ROS play a major role in the induction of DNA damage, which is why changes in the expression of genes that were involved in the body’s response to oxidative stress were observed in experimental groups, which indicates abnormalities in erythrocyte nucleus formation. Biomarkers of oxidative stress that are often used in toxicology experiments include superoxide dismutase (SOD), glutathione peroxidase (GPX), glutathione reductase (GSR), or catalase [44,45]. These enzymes function at different levels during the body’s response to oxidative stress. Glutathione reductase and glutathione peroxidase are responsible for maintaining the homeostasis of reduced glutathione—the most important substrate for the synthesis of bioproteins—as well as ascorbic acid and α-tocopherol, the main antioxidants. Dismutase, on the other hand, is responsible for the deactivation of free radicals in the body [44]. Meanwhile, catalase alleviates oxidative stress by destroying cellular hydrogen peroxide to produce water and oxygen [45]. After a one-week exposure of zebrafish to the tested xenobiotics, a reduction in the expression levels of the *cat*, *gpx1a*, and *sod1* genes was observed in all test groups compared with the controls. Similar effects of silver nanoparticles on oxidative stress markers in the liver of fish were observed by other authors. Valerio-García et al. [46] observed a reduction in the activity of the SOD, CAT, and GPX enzymes after exposure to AgNPs in the liver of the goodeid fish (*Chapalichthys pardalis*), a species endemic to Mexico. Furthermore, in the liver of medaka fish, Wu and Zhou [47] found a dose-related decrease in the activity of antioxidant enzymes such as SOD, CAT, and GPX. However, the level of *gsr* expression was higher in groups with low concentrations of AgNPs (AgNP 0.01 and 0.05 mg/dm^3^) than in the control group, while it was lower in the AgNP 0.1 and 1.0 mg/dm^3^ groups. A similar pattern of GSR activity was observed in carp (*Cyprinus carpio*) liver, where at lower concentrations of AgNPs, activity was higher compared with the control group; yet, in the groups with the highest concentration of the xenobiotic, the GSR activity decreased compared with the control group, as observed by Kakakhel et al. [48]. Based on these results, the effect of AgNPs on the formation of oxidative stress and the subsequent changes of genotoxic nature associated with DNA damage is indisputable. However, the very link between particle properties and ROS generation is still troublesome to trace in nanoecotoxicology and requires further studies of the toxicity mechanisms. Moreover, as Skjolding et al. [41] pointed out, there are many pathways linking cytotoxicity, genotoxicity, and oxidative stress with ROS generation, therefore it is difficult to identify specific pathways activated by particular nanoproducts.

Another issue under investigation is the role of the CYP1A protein in modulating the body’s response to silver nanoparticle exposure. In the current study, the AgNP and AgNO_3_ exposure had no effect on the *cyp1a* expression. A similar lack of a relationship between exposure to silver nanoparticles and the *cyp1a2* gene expression in rainbow trout livers was shown by Scown et al. [49]. This may indicate a lack of strong stimulation of the phase I biotransformation during the detoxification of organs exposed to aqueous solutions of silver nanoparticles.

## 4. Materials and Methods

The experiment was conducted in accordance with permission number 70/2015 from the 3rd Local Ethics Commission regarding Experiments in Animals at the Warsaw University of Life Sciences. The subjects were sexually mature zebrafish (*Danio rerio*) of both sexes of the wild strain AB/TU, (male 32.3 ± 0.1 mm; female 36.7 ± 0.4 mm). The fish originated from the Zebrafish Core Facility of the International Institute of Molecular and Cell Biology in Warsaw.

### 4.1. Schematic of the Experiment

Before the experiment, the fish in the aquaria underwent a seven-day acclimatization. The toxicity test was maintained in glass tanks with a capacity of 5 dm^3^, at a density of 1 specimen per 1 dm^3^. During the exposure, males and females were kept in separate aerated tanks at 26 °C. The water was prepared following the recommendations for the maintenance of this species in livestock/breeding facilities. According to Williams and Renquist [50], the E3 culture solution, a ×60 concentrate contained: 17.4 g of NaCl, 0.8 g of KCl, 2.9 g of CaCl_2_ × 2H_2_O, and 4.89 g of MgCl_2_ × 6H_2_O dissolved in 1 dm^3^. The solution was then adjusted to a pH of 7.2 using NaOH and HCl. Fish were exposed to the xenobiotic water solutions for 7 days. In the experiment, the following research groups were established: a control group (without exposure to xenobiotics), a group exposed to silver ions at a concentration of 0.01 mg/dm^3^ (AgNO_3_ group), groups exposed to silver nanoparticles (AgNP groups) at five different concentrations: 0.01, 0.05, 0.1, 0.5, and 1.0 mg/dm^3^. Each group had six repetitions. The experiment took the form of a static renewable exposure. Half of the volume of water in the tanks was changed every 24 h, and the level of xenobiotics was supplemented to the initial concentration. Water parameters were maintained at the following levels: the total hardness (GH) was between 142.4 and 249.2 mg/dm^3^; carbonate hardness (KH) was between 106.8 and 178 mg/dm^3^; concentration of NO_3_^−^ was less than 10 mg/dm^3^; concentration of NO_2_^−^ was 0 mg/dm^3^; and pH was at 7.6 ± 0.2, which is in accordance with the requirements of the examined species. The water parameters were controlled with commercial aquarium water analysis kits (Tetra, Germany). During the acclimatization and experimental phases, fish were fed twice a day ad libitum using commercial feed (Zebra-feed, Sparos, Portugal) (at 9 am), and frozen *Artemia* (at 4 pm).

After the exposure was completed, spawning was carried out to see if the tested xenobiotics affected the reproductive desire of the fish. The spawning process was necessary so that there were oocytes in the fixed material formed in the process of meiosis in the gonads after the onset of exposure to xenobiotics. Spawning was carried out in the water with parameters corresponding to the tested groups. The spawning of zebrafish was stimulated by photoperiod and separation of fish by sex according to the recommendations for breeding this species. It was conducted in the morning, after a 10-h period of darkness. At the end of the experiment, survival rates were assessed, and then 15 males and 15 females from each group were anesthetized with MS-222 solution (tricaine methanesulfonate, 3-amino-benzoic acid ethyl ester, Sigma-Aldrich, St. Louis, MO, USA) and decapitated. The material was collected for the histological (light and electron microscopy), genetic, and genotoxicity analyses.

### 4.2. Origin and Characterization of Silver Nanoparticles and Silver Nitrate

**Figure 8:**
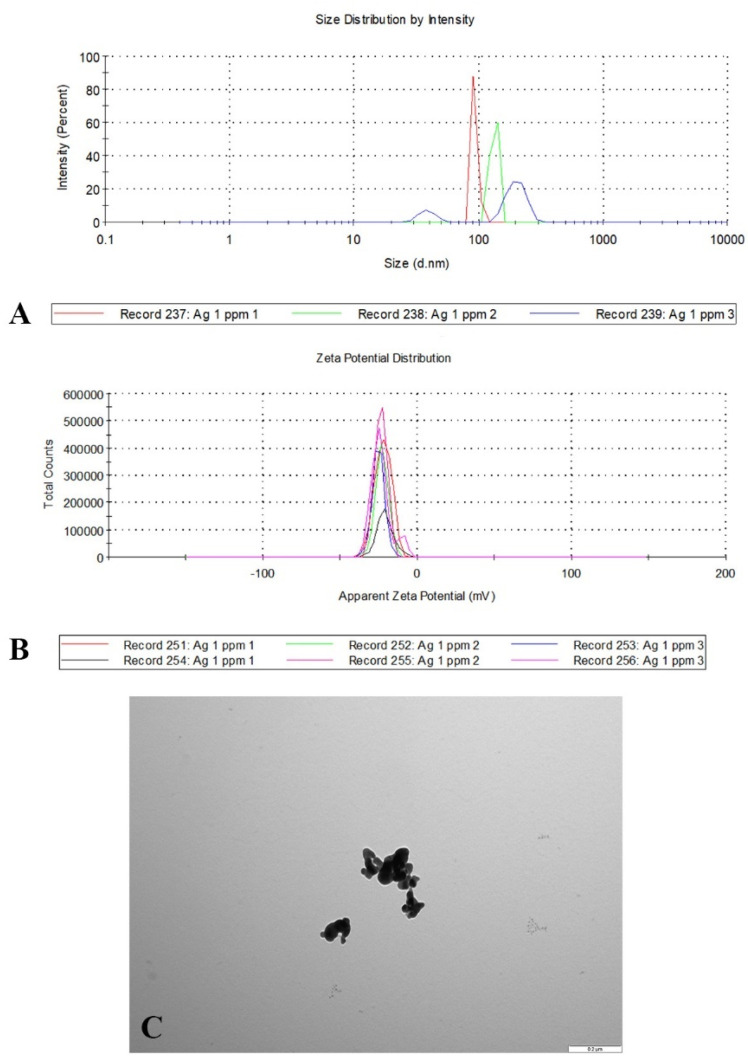
The parameters and shape of the silver nanoparticles used in the experiment: (**A**) size distribution, (**B**) zeta potential distribution (mV), and (**C**) morphology of silver nanoparticle aggregates. Scale bar: 0.2 µm.

PvP (polyvinylpyrrolidone)-coated silver nanoparticles (AgNPs) (Sigma Aldrich, Darmstadt, Germany, catalog number 576832—5G; diameter was less than 100 nm) were used in the study. They were suspended in water at a concentration of 1000 mg/dm^3^ and then subjected to sonification, consisting of 3 cycles lasting 15 min each using an ultrasonic cleaner Ultron U-505 (Ultron, Dywity, Poland) at 45 °C. A coating made with polyvinylpyrrolidone (PvP) was used to slow down the agglomeration process and ion release during the experiment. This type of coating stabilizes silver nanoparticles regardless of the ionic strength, pH, and electrolyte compounds of the water [23]. The solution of silver ions was obtained by dissolving silver nitrate salts in distilled water (Poch, Gliwice, Poland; AgNO_3_, nr. 814322777). To characterize nanoparticles in aqueous solutions, a dynamic light scattering (DLS) analysis was performed. Observations of nanoparticles and nanoparticle agglomerates were made under a transmission electron microscope. A DLS analysis was performed to determine the average hydrodynamic diameter of the AgNPs, which ranged from 39 nm to 93 nm, but the agglomerates were more than 202 nm in diameter. The zeta potential for all tested concentrations was similar; the mean was −23.4 mV (Figure 8). Both variables were measured using a Zetasizer Nano S90 analyzer (Malvern Instruments Ltd., Malvern, UK). We recorded the nanoparticle size and zeta potential analyses using DLS and was performed at room temperature (25 °C). Each measurement was repeated three times. The analysis and microscopic measurements showed the presence of nanoparticles suspended in the solution with an average diameter of 42.10 ± 19.27 nm (min. 6.14 nm; max. 108.79 nm) and nanoparticle agglomerates with diameters above 200 nm (Figure 7). All analyses and measurements in transmission electron microscopy were performed using a FEI 268D “Morgagni” transmission electron microscope (FEI Company, Hillsboro, OR, USA) equipped with an Olympus-SIS “Morada” digital camera (Olympus, Münster, Germany).

### 4.3. Histology Analyses

The decapitated fish taken for analysis (10 individuals from each experimental group) were fixed in Bouin’s solution. Then, the fish were dehydrated in an increasing range of ethanol concentrations, cleared in xylene, and embedded in paraffin. The research material was cut to be 6 μm thick using the Leica RM 2265 microtome (Leica Microsystems, Wetzlar, Germany). The obtained slides were stained with hematoxylin and eosin (HE) to assess the morphology of the gonads and liver. To determine the proliferative potential of the liver, the slides were stained using the immunohistochemical method. To detect proliferating hepatocyte nuclei, the PCNA (proliferation cell nuclear antigen) monoclonal mouse antibody, clone PC10 (cat no. M0879; DAKO, Glostrup, Denmark) was used, according to the methodology described by Kamaszewski et al. [51]. The following histomorphometric measurements (100 measurements from 1 individual) were taken in the liver on the HE-stained preparations: the area of the hepatocytes (µm^2^), area of the hepatocyte nuclei (µm^2^), and area of lipid vacuoles in hepatocytes (µm^2^). On slides stained with anti-PCNA antibodies, the proliferation index expressed as a percentage of PCNA-positive nuclei to hepatocyte nuclei was analyzed on slides stained with anti-PCNA antibodies on 10 sections of the liver from each individual. Sections throughout the male gonads were analyzed for the percentage of spermatogonia, spermatocytes, and spermatids in the 25 seminal tubules of 5 individuals from each experimental group. Meanwhile, in the ovaries of all females (*n* = 5) subjected to the histological analysis, we measured the diameter distribution of oocytes in the ovaries. The degree of oocyte maturity was assessed based on the study by van der Ven and Wester [52]. The following classes were distinguished based on the diameter of the female germ cells and their morphology: (1) oogonia (0–20 µm diameter); (2) previtellogenic oocytes (covering stages 1, 2, and 3; 20–160 µm diameter); (3) vitellogenic oocytes (covering stages 4, 5, and 6; 160–400 µm diameter); and (4) growth and maturation stage (covering stage 7; 400–800 µm diameter). All histomorphometric measurements were taken for the 5 individuals from each experimental group using a Nikon Eclipse 90i microscope (Tokyo, Japan) in tandem with a Nikon DS5-U1 camera (Tokyo, Japan) and the image analysis program NIS ELEMENTS (Tokyo, Japan).

### 4.4. Ultrastructure Analyses of Hepatocytes

The analyses of hepatocyte ultrastructure were performed on liver fragments which were fixed in 2% (*w*/*v*) of paraformadehyde and 2.5% (*v*/*v*) of glutaraldehyde solution in a 0.05 M cacodylate buffer (pH 7.2) for 2 h. The samples were rinsed 4 times with cacodylate buffer (pH 7.2) at the same concentration. They were then post-fixed for 1 h at 4 °C in a 2% (*w*/*v*) solution of osmium tetroxide dissolved in a 0.05 M cacodylate buffer. The samples were dehydrated in increasing concentrations of ethanol (from 10 to 100%) and finally substituted with pure propylene oxide. They were infiltrated with an epoxy resin of medium hardness (resin composition for 100 mL: 45.6 mL of glycid ether, 30.8 mL of DDSA, 23.5 mL of MNA, and 1.5 mL of DMP-30). The resin was polymerized for 24 h at 60 °C in silicone flat embedding molds. The ultra-thin (80 nm thick) sections for transmission electron microscopy analysis were made using the Leica UCT ultramicrotome (Leica Microsystems, Wetzlar, Germany). The material was contrasted in 1.2% (*w*/*v*) of uranyl acetate solution (Sigma-Aldrich, St. Louis, MO, USA) for 10 min and saturated lead citrate solution (Sigma-Aldrich, St. Louis, MO, USA) for 10 min. Sections were examined on an FEI 268D “Morgagni” transmission electron microscope (FEI Company, Hillsboro, OR, USA) equipped with an Olympus-SIS “Morada” digital camera (Olympus, Münster, Germany).

### 4.5. Genotoxicity Analysis

The genotoxic effects of the tested xenobiotics were analyzed using a micronucleus assay. Two blood smears were made from each of the 10 fish (5 of each sex) collected for the histological analysis, which were fixed in absolute methanol for 10 min, then stained for 40 min with a freshly prepared 10% Giemsa solution. The analysis of the slides was carried out according to the methodology described by Vignardi et al. [53]. The images of the blood smears were analyzed by light microscopy under 1000× magnification. For the morphometric analysis, 1000 erythrocytes were randomly observed to obtain the frequency (%) of micronuclei (MN) and other erythrocyte nuclear abnormalities (ENA), such as a reniform nuclear (R), which has a kidney-like shape and segmented nuclear (S), in which the nucleus is separated by a constriction into parts that are not necessarily the same size.

### 4.6. Gene Expression Analysis

To analyze the expression of genes of oxidative stress markers: catalase (*cat*), glutathione peroxidase (*gpx1a*), glutathione reductase (*gsr*), superoxide dismutase (*sod1*), and the phase I of biotransformation, cytochrome P450 1A (*cyp1a*), the material was fixed in RNAlater (catalog no. R0901-100ML; Sigma-Aldrich, Poznań, Poland) and then the total RNA from the livers of 10 fish was isolated using the NucleoSpin RNA/Protein kit (Macherey-Nagel, Germany). The quantity and the quality of the obtained RNA were verified using a spectrophotometric measurement of the optical density using a NanoDrop ND-1000 spectrophotometer (Thermo Fisher Scientific, Waltham, MA, USA) at wavelengths of 260 nm, 280 nm, and 230 nm and electrophoresis in 1% agarose gel. Then, the obtained RNA was transcribed into cDNA using a Maxima™ First Strand cDNA Synthesis Kit for RT-qPCR reverse transcription kit (Thermo Fisher Scientific, Waltham, MA, USA). The obtained and purified genetic material was used in the real-time PCR reaction which was performed using a FIREPol **^®^** HOT EvaGreen qPCR Mix **^®^** Plus kit (Solis Biodyne, Tartu, Estonia). The proper temperature profile for each gene was chosen after the optimization using the standard protocol: 95 °C for 15 min for HOT FIREPol**^®^** DNA Polymerase activation, 40 cycles at 95 °C for 5–10 s for denaturation, 60 °C for 10–30 s for annealing, and 72 °C for 15–30 s for extension. The primers of the genes studied related to biotransformation and antioxidant protection, *gsr*, *cat*, *gpx1a*, *sod1*, and *cyp1a*, are presented in Table 2. The PCR reactions were carried out using a RotorGene Q thermocycler (Qiagen, Germantown, MD, USA) using the RotorGene Q software (Qiagen, Germantown, MD, USA). To provide a relative expression, the results obtained for the examined genes were normalized to the reference gene chosen from among 3 genes: actin beta (*actb*), beta-2-microglobulin (*b2m*), actin beta 2 (*actb2*) using the NormFinder program (https://moma.dk/normfinder-software; accessed on 2 July 2020) for the identification of the optimal normalization gene. The results are presented in arbitrary units; the ratio of the target gene expression to the expression of the reference gene with a control group was calculated as 1.

### 4.7. Statistical Analysis

The statistical analysis was carried out in the STATISTICA 13.0 software(Tibco Software, Palo Alto, CA, USA). Spearman’s rank test (*p*-value < 0.05) was used to indicate differences between the toxicity biomarkers. To indicate the differences for each of the parameters tested, the compliance with normal distribution was first checked by Shapiro–Wilk tests. Parameters with inconsistent normal distributions were standardized by bypassing data to the logarithmic scale. A single-factor analysis of the ANOVA variance was used, supported by Tukey’s post hoc test. The results were displayed as the mean value ± SD (standard deviation). Only the results of the gene expression were expressed as the mean value ± standard error of mean (SEM).

## 5. Conclusions

Based on the research results obtained, it can be concluded that the toxicity of AgNPs does not have to be directly proportional to the examined concentration of this nanoxenobiotic, especially in concentrations corresponding to those observed in the environment. Most of the toxicological tests show an increase in negative effects with increased AgNP concentration. Based on the results, it can be concluded that even a one-week exposure of zebrafish to silver nanoparticles causes cytotoxic changes, activates the immune system (phagocytic activity of liver macrophages), negatively affects the meiosis process in the ovaries and testes, and generates oxidative stress. This holistic analysis of the fish body’s response to exposure of low, environmentally-referenced AgNP concentrations leads to the conclusion that even a short-term exposure to nanoxenobiotics may lead to reproductive disorders and induce abnormalities in the demographic structure of populations.

## Figures and Tables

**Table 1:** The percentage of micronuclei and other abnormalities in the erythrocyte morphology from the blood smears of fish from the experimental groups. Mean values ± SD are shown.

Parameters	Experimental Groups						
	Control	AgNP 0.01	AgNP 0.05	AgNP 0.1	AgNP 0.5	AgNP 1.0	AgNO_3_ 0.01
Percentage of micronuclei (%)	0 ± 0	0 ± 0	0 ± 0	0 ± 0	0 ± 0	0.1 ± 0.04	0 ± 0
Percentage of segmented erythrocytes (%)	0.15 ± 0.04	0.15 ± 0.04	0.3 ± 0.06	0.2 ± 0.04	0.45 ± 0.07	0.75 ± 0.08	0.3 ± 0.04
Percentage of reniform erythrocytes (%)	0 ± 0	0.05 ± 0.02	0.05 ± 0.02	0 ± 0	0.05 ± 0.02	0 ± 0	0.05 ± 0.02

**Table 2:** The sequences of the genes studied and the reference genes.

Gene Symbol (GenBank Accession Number)	Primer Sequence		Product Length
	Forward	Reverse	
*actb* (*NM_131031*)	CGAGCAGGAGATGGGAACC	CAACGGAAACGCTCATTGC	102
*b2m* (*NM_131163.2*)	GCCTTCACCCCAGAGAAAGG	GCGGTTGGGATTTACATGTTG	101
*actb2* (*NM_181601.5*)	ACGATGGATGGGAAGACA	AAATTGCCGCACTGGTT	94
*gsr* (*NM_001020554.1*)	TGTGCCAGGATCCAGTTTAGG	GCACCCCTCCTTGTCGTATG	167
*Cat* (*NM_130912.2*)	CTCCTGATGTGGCCCGATAC	ATCAGGTTTTGCACCATGCG	172
*gpx1a* (*NM_001007281.2*)	GCACCAGGAGAACTGCAAGAATG	CAGAGGGTGGGCGTTTTCAC	130
*sod1* (*NM_131294.1*)	TGGTGACAACACAAACGGCT	TCTCCGACGTGTCTCACACTA	99
*cyp1a* (*NM_131879.2*)	GGAGCCGGTTTCGACACTAT	GTGCGATCCTTCCCGATCTT	122
